# Measuring and understanding social-emotional behaviors in preschoolers from rural Pakistan

**DOI:** 10.1371/journal.pone.0207807

**Published:** 2018-11-27

**Authors:** Jenna E. Finch, Aisha K. Yousafzai, Muneera Rasheed, Jelena Obradović

**Affiliations:** 1 Department of Psychology, University of Nebraska-Lincoln, Lincoln, Nebraska, United States of America; 2 Department of Global Health and Population, T.H. Chan School of Public Health, Harvard University, Cambridge, Massachusetts, United States of America; 3 Department of Paediatrics and Child Health, Aga Khan University, Karachi, Pakistan; 4 Graduate School of Education, Stanford University, Stanford, California, United States of America; TNO, NETHERLANDS

## Abstract

The Strengths and Difficulties Questionnaire (SDQ) is widely-used to measure symptoms of common childhood behavioral problems that may lead to mental health difficulties. In a sample of 1,302 highly-disadvantaged mothers and their preschoolers, we evaluated the factor structure and reliability of the parent-report version of the SDQ in rural Pakistan. Confirmatory factor analyses suggested that the original structure of the SDQ was not appropriate for our data. We created conceptually- and empirically-coherent measures of children’s externalizing behavior problems and prosocial skills. Child and family correlates of social-emotional behaviors were similar to those found in other countries, supporting the validity of our new composites. Girls and children with more siblings had fewer externalizing behavior problems and more prosocial behaviors at four years. Further, maternal depressive symptoms and food insecurity were uniquely linked to more externalizing behavior problems at four years. In contrast, maternal education, home environment quality, and social-emotional skills at two years were associated with more prosocial behaviors at four years.

## Introduction

Children’s abilities to get along with their peers, control their behaviors, and avoid distractions are crucial for their social and academic success [1]. Yet, over a quarter of preschoolers in low- and middle-income countries (LMIC) demonstrate low social-emotional skills [2]. Children who display exhibit behavioral problems in early childhood are more likely to have mental health challenges later in life [3]. Mental health problems are part of the intergenerational poverty cycle and implicated in poor educational and employment outcomes [4]. It is important to study the development of social-emotional behaviors in early childhood, so that we can understand how to promote these behaviors when children’s development is most malleable [5]. Still, most research in LMIC focuses on young children’s cognitive and physical development [6–8]. This is partially due to the difficulty in measuring social-emotional behaviors, particularly for young children not enrolled in school.

This study describes the adaptation and use of a parent-report of preschoolers’ behavior on the Strengths and Difficulties Questionnaire (SDQ; [9]), in rural Pakistan. Pakistan is a highly-disadvantaged society with similar problems to other LMICs such as low access to schooling and high rates of illiteracy and poverty [10,11]. There are unique challenges in assessing young children’s social-emotional behaviors in disadvantaged settings where teacher report is not feasible due to low rates of preschool attendance [12]. In rural LMIC contexts where few children attend preschool, researchers must rely on parent-report of children’s social-emotional behaviors. When parents have low literacy levels and are not familiar with Likert-scale assessments it is particularly difficult to get reliable reports of children’s behaviors. It is important to use an instrument that is accessible to parents and simple to administer by local community members. Advancing this area of research is crucial as we seek to reduce mental health problems and advance the school readiness skills of children in LMIC.

### Preschoolers’ social-emotional behaviors in low- and middle-income countries

Researchers have generally translated Western measures of social-emotional behaviors for use in LMIC without examining whether these measures are appropriate in the context. Simply translating measures is problematic because certain behaviors may be considered normative in one context but not another. Further, societal expectations for children’s behaviors may differ across countries and ethnic groups. For example, in Western countries children are encouraged to initiate and maintain social activities, whereas social initiation is not as highly valued in group-oriented cultures because it may undermine group cohesiveness [13]. Finding appropriate measures of children’s social-emotional skills in LMIC is essential, as we know very little about the developmental change and stability of social-emotional behaviors in these settings. In high-income contexts, developmental trajectories of social-emotional behaviors from infancy through adolescence are important predictors of long-term outcomes [1,3].

In Pakistan, parental reports of children’s behavioral problems are much higher than those reported in higher-income countries [14,15]. Country-level prevalence estimates show that over a quarter of preschoolers in Pakistan have low social-emotional skills, as indexed by aggressive behavior, high levels of distraction, and poor social competence on the Early Childhood Development Index (ECDI; [2]). This measure of early development uses three questions to assess children’s social-emotional skills, and thus provides only broad information about children’s behaviors used to track country-level estimates of children’s wellbeing over time as part of UNICEF’s Multiple Indicator Cluster Surveys. Use of more nuanced measures are needed to provide a complete picture of children’s social-emotional behaviors in this context.

### The strengths and difficulties questionnaire

Children’s social-emotional behaviors encompass their abilities to experience, express, and manage their emotions and develop positive relationships with children and adults [16]. While social-emotional development includes a broad range of skills and beliefs, the SDQ focuses on easily observable behaviors that are related to common childhood mental health symptoms. The SDQ is a relatively short 25-item behavioral screener and has been used widely for clinical, epidemiological, and intervention research. The SDQ includes positively worded items (e.g. “shares readily with other children”) and is free of cost, making it an appealing measure for researchers. Goodman’s original version of the SDQ has five subscales, four focused on children’s behavior problems (Peer Problems, Hyperactivity-Inattention, Emotional Problems, and Conduct Problems) and one capturing children’s Prosocial Behaviors. The original five-subscale structure of the SDQ has been validated and demonstrated adequate reliability in many wealthier countries, such as Spain, Denmark, Norway, and Japan [17–19].

Most studies in LMIC have not examined the structure and reliability of the SDQ subscales (e.g. [20–22]). The limited number of studies that explored the structure of the SDQ in LMIC found that the items did not load onto the original five subscales [23,24]. For example, Thabet and colleagues used confirmatory factor analyses and chi-square difference tests to compare whether each of the original subscales was better fit by a one- or two-factor model [24]. For all five subscales, they found that the two-factor subscale model fit better, suggesting the original one-factor subscales model did not fit the data. They noted that several items did not load as highly onto the original subscales as they did in wealthier samples. Further, they found low reliability (ranging from .182 to .654) for the subscales [24]. They never used exploratory factor analysis to examine whether a different structure across all SDQ items better fit their data.

An Urdu translation of the SDQ has been used in Pakistan and demonstrated evidence for both construct and discriminant validity. This prior work was completed in an urban setting (Karachi) using children ages 4 to 16 [15,25,26]. The validation studies demonstrated the SDQ was able to adequately discriminate between children who had received clinical psychiatric diagnoses and those who did not have clinical-level behavioral problems [25]. Further, SDQ scores were significantly correlated with parent-report of behavioral problems on the Child Behavior Check List (CBCL) in primary school children [26]. No studies in Pakistan, to our knowledge, have explored the structure or reliability of the parent-report version of the SDQ. Researchers in Pakistan have used the original five subscales to measure children’s social-emotional behaviors without reporting reliability statistics [25–28]. In summary, there is little evidence to suggest that the original structure of the SDQ provides reliable measures of children’s behaviors in LMIC. More research is needed to understand which items cohere as reliable measures of children’s social-emotional behaviors in highly-disadvantaged contexts.

### Child- and family-level correlates of social-emotional behaviors in rural Pakistan

Poverty, and the broader socio-cultural context, work through two pathways to impact children’s development in LMIC [5,6]. First, poverty and associated risk factors are linked to increased biological risks, including poor growth and nutrient deficiencies, later affecting children’s development [6]. Second, poverty is linked to poor parenting practices and maternal depression, which then impact children’s development. It is important to explore socioeconomic indices, biological risk factors, child-level factors, and family experiences together to provide a complete picture of how children’s environments influence their development.

There are only a couple of studies that examined socioeconomic differences in Pakistani children’s social-emotional behaviors. One study found that parental education, but not family wealth, was linked to fewer behavioral symptoms on the SDQ, as indexed by higher scores on the original four behavior problem subscales, in a sample of lower-income primary school children ages 5 to 11 [15]. In contrast, Maselko and colleagues [28] found that low socioeconomic status, but not maternal education, was linked to higher scores on the Emotional Problems subscale and lower scores on the Prosocial Skills subscale in primary school students. They did not find any socioeconomic differences on the Conduct Problems, Hyperactivity-Inattention, or Peer Problems subscales.

There is some evidence that child-level factors, such as gender and biological risk, are linked to children’s social-emotional development in Pakistan. Similar to developed countries, male children show higher rates of behavioral problems and lower rates of prosocial skills in Pakistan [14,15,28]. In a sample of low-income primary school students, nearly half of the boys had clinical levels of behavior problems on the SDQ [14]. Girls, however, had higher levels of Emotional Problems in a rural sample of Pakistani seven-year-olds [28]. Further, measures of height and weight are markers of early malnutrition, deprivation, and biological risk in LMIC. In a sample of orphaned Pakistani children, low weight-for-height and malnourishment were associated with more Conduct Problems on the SDQ [27]. Using nationally-representative data, Miller and colleagues [29] found that low height-for-age was negative associated with social-emotional development on the ECDI in Pakistan.

Finally, different aspects of children’s home environments affect children’s social-emotional development. First, there is strong evidence that maternal depressive symptoms are associated with children’s social-emotional behaviors and mental health in LMIC [30–32]. In Pakistan, school-age children of depressed mothers had more behavioral symptoms on the SDQ, compared to children with non-depressed mothers [28]. Second, higher quality home environments, including responsive parenting and cognitively-stimulating learning activities, have been associated with higher scores on a composite measure of children’s cognitive, social-emotional, and physical development [33]. There has been no research in LMIC, to our knowledge, examining the effects of the home environment on children’s social-emotional development specifically. Third, siblings may provide preschoolers with opportunities to practice and improve their social-emotional skills before they enter school [34]. In general, there is little evidence about which early risk factors are most salient for children’s social-emotional development in LMIC [35]. Exploring these pathways simultaneously, will allow for comparison between socioeconomic, biological, child-level, and family-level risks to evaluate which experiences are the most impactful for preschoolers’ social-emotional development in LMIC.

### Current study

The current study evaluates the reliability and validity of the SDQ for a sample of highly-disadvantaged preschoolers in Pakistan enrolled in a randomized control trial with responsive stimulation and nutrition interventions. First, we discuss the importance and challenges of using parental report to measure children’s social-emotional behaviors in LMIC. Second, we explore the reliability and factor structure of the SDQ items, to assess whether the original subscales apply to this population. If the original structure does not fit our data, we will use exploratory factor analysis to find the most appropriate way to composite items in our sample. Third, we test developmental continuity in children’s social-emotional behaviors from ages two to four years. Finally, we explore child-level and family-level correlates of Pakistani preschoolers’ social-emotional behaviors to provide evidence for the construct validity of the SDQ in this context and to identify factors that correlate with children’s social-emotional behaviors. By including multiple indices of children’s home environments, this study elucidates which specific aspects of children’s early experiences are most predictive of social-emotional behaviors for Pakistani preschoolers.

## Method

### Participants

The sample comprised 1,302 children (46% girls) and primary caregivers (99% mothers) who were enrolled in the Pakistan Early Child Development Scale-Up (PEDS) Trial, registered with ClinicalTrials.gov, #NCT007159636. The PEDS trial was a community-based, cluster-randomized controlled trial of early responsive stimulation and nutrition interventions from birth to two years [36]. Informed consent was obtained from all participants. Parents either signed a consent form or provided an ink thumbprint to indicate their consent, which is a common practice in contexts with high illiteracy rates. The current study represented a longitudinal follow-up of the birth cohort at four years (*M* age = 4.03, *SD* age = 0.03, age *range* = 3.75–4.27). Ethical approval for the longitudinal follow-up study was obtained from the ethical review committee of the Aga Khan University, Karachi, Pakistan. Attrition at the follow-up (*N* = 187, 12.56%) was predominantly due to disabilities, deaths, and migration.

Participants resided in the predominantly agricultural Naushero Feroze District, in Sindh Province, Pakistan. The sample was exposed to high levels of adversity. Monthly household income averaged $100 USD (*SD* = $140 USD) and 68% of mothers and 31% of fathers were illiterate. At baseline, mothers were, on average, 28 years of age (SD = 5.88 years) and reported an average of 2.26 years of education (*SD* = 3.77). The average number of children per mother was 4.2 (*SD* = 2.2). When children were 24 months, one third of families reported food insecurity and a substantial proportion of children were underweight (43%) and exhibited stunting (61%).

### Procedures

A birth-cohort of children, born between April 1, 2009 and March 31, 2010, was invited to enroll in the PEDS trial with their primary caregivers. Data were collected at baseline, during the PEDS Trial (when children were 12 and 18 months old), at the end of the PEDS trial (when children were 24 months old), and at the 48-month follow-up. Data were collected during home visits by two community-based data collectors who received extensive training on ethics, interacting with families, understanding the evaluation constructs, administering measures, and dealing with assessment barriers. The second assessor engaged other family members to ensure that mothers had privacy during the interviews. All questionnaires and child assessments were administered in the local language, Sindhi.

### Measures

Table 1 shows descriptive statistics for all study variables.

**Table 1:** Descriptive statistics for all study variables.

	*N*	Mean / %	SD	Skew
Externalizing behavior problems	1298	0.951	0.523	0.058
Prosocial behaviors	1298	1.529	0.363	-0.863
Male child	1302	53.92%		
Maternal education	1302	2.192	3.686	1.591
Family wealth	1294	-0.002	0.988	1.446
Number of siblings	1267	2.5	2.309	1.085
Responsive stimulation intervention	1302	50.59%		
Enhanced nutrition intervention	1302	48.08%		
Maternal depressive symptoms (12m)	1267	6.605	4.462	0.559
Home environment quality (18m)	1273	30.811	5.444	-0.087
Food insecurity (24m)	1301	32.78%		
Height-for-age (24m)	1278	-2.333	1.123	-0.153
BSID-III social-emotional	1297	93.709	18.334	0.464
BSID-III cognitive-language	1298	80.5	12.793	0.169

### Children’s social-emotional behaviors

The parent-version of the SDQ [9] was used to measure children’s social-emotional behaviors. The SDQ contains 25 questions about different positive and negative aspects of the child’s behavior. Responses are made on a three-point Likert scale; “*not true*”, “*somewhat true*”, and “*certainly true*.” An Urdu translation of the SDQ has been previously validated and used in Pakistan [25,26]. For our study, the Urdu version was translated into Sindhi and then independently back-translated to English. We began with an Urdu version of the SDQ because it was previously adapted for the local context, including important cultural modifications of item phrasing. In addition, Urdu is linguistically more similar to Sindhi than English. Through discussions with community members and a multi-disciplinary research team, slight adjustments were made to ensure mothers would understand the questions. For example, complex formal words were replaced with simpler local terms. The measure was pilot tested with 25 mothers of preschoolers drawn from the study area (but not in the study) before it was finalized (see the S1 Appendix for the full adaptation process).

The SDQ was read aloud to mothers, given the low literacy rates in the sample and a conversational approach was employed. Community-based assessors provided behavioral examples and probes when the question meaning was unclear. The examples and probes were standardized across assessors and included on the questionnaire form (see Table A in S2 Appendix, for all probes/examples). For example, for the item “*easily distracted*, *concentration wanders*,” assessors would follow up with the probing question: “if you are speaking to CHILD does s/he listen to what you are saying or does s/he lose interest?” Mothers verbally responded to the items and the assessor scored the responses. Inter-rater reliability, conducted concurrently in a single visit, was very high (Bland-Altman reliability agreement scores ranged from 0.964 to 0.986 for the different subscales). Further, 7-day test-retest reliability conducted with 25 mothers during the pilot study was high (0.94 to 1.00 for different subscales, see the S1 Appendix for more detail). The final SDQ items are presented in Table A in the S2 Appendix and correlations among all SDQ items are presented in Table B in the S2 Appendix.

### Maternal depressive symptoms

*Maternal depressive symptoms* were measured using a translated and validated version of the Self-Report Questionnaire (SRQ-20; [37]) at 12-months postpartum. Twenty items used to assess the presence of symptoms in the past four weeks (e.g., “*Is your appetite poor*?”, *“Do you feel nervous*, *tense*, *or worried*?*”*, *“Do you feel unhappy*?*”*) were summed to create a total score (*M* = 6.60, *SD* = 4.46, range = 0–19).

### Home environment quality

Home environment quality at 18 months was measured using the adapted infant/toddler version of the Home Observation for Measurement of the Environment Inventory (HOME; [38]). The original items were adapted following extensive piloting, by adding culturally relevant examples and by providing specific observation guidelines for ratings of environment and maternal behaviors during the home visit. There were six dimensions: (1) responsivity, (2) acceptance, (3) organization, (4) learning materials, (5) involvement, and (6) variety. Items were scored based on mothers’ report of family living patterns and habits, observation of spontaneous mother-child interactions, and orderliness and enrichment potential of the physical home environment. A measure of *home environment quality* was generated by summing all 45 items (*N* = 1273, *M* = 30.81, *SD* = 5.44, α = .82).

### Food insecurity

A binary measure of *food insecurity* (1 = *food insecure*) represented the lack of access of safe and nutritionally adequate food at 24 months ([39]; 24 months: *M* = 0.33, *SD* = 0.47; 48 months: *M* = 0.37, *SD* = 0.48).

### Height-for-age

Trained assessors measured child’s height at 24 months to the nearest 0.1 cm in accordance with standardized guidelines. Height was converted into a standardized *height-for-age* index using WHO Anthro software V3.2.2 (*M* = -2.33, *SD* = 1.12, range = -6.63–1.11). Low height-for-age reflects chronic malnutrition as values below -2.00 represent stunted growth.

### Early cognitive, language, and social-emotional skills

Children’s cognitive, language, and social-emotional skills at 24 months of age were assessed using the Bayley Scales of Infant and Toddler Development, Third Edition (BSID-III; [40]). The BSID-III was translated through a rigorous process including quality assurance checks using back translation. A local team of experts carefully reviewed the BSID-III items to ensure they were culturally appropriate, followed by field testing and rigorous training. The cognitive composite score (*M* = 78.24, *SD* = 14.61) measured children’s sensorimotor skills, exploration and manipulation of the environment, object relatedness, and memory. The language composite score (*M* = 82.76, *SD* = 13.43) measured children’s receptive and expressive communication skills, including pre-verbal communication, vocabulary development, and verbal comprehension. Both the cognitive and language scales were evaluated through direct assessments with children. The cognitive and language composite scores were averaged to create a measure of *cognitive and language skills* (*M* = 80.50, *SD* = 12.79, *r* = .66).

The *social-emotional* composite score (*M* = 93.71, *SD* = 18.33) measured children’s early play behaviors, communication of needs, use of emotional signals to solve problems, and responsiveness to people and the environment. Parents reported on these items in an interview setting using a conversational approach, similar to the SDQ. There was some conceptual overlap between the SDQ and the BSID-III social-emotional scale. For example, items that assessed early precursors of social behaviors (i.e. telling parent what s/he wants with one or a few words; copies or imitates make-believe play; seems happy when s/he sees a favorite person; responds to people talking or playing with him/her) were conceptually related to items from the original SDQ Prosocial and Peer Problems subscales (i.e. helpful if someone is hurt, upset, or feeling ill; kind to younger children; generally liked by other children). However, the BSID-III social-emotional composite score does not capture early indicators of problem behaviors such as emotional dysregulation or difficult temperament.

### Intervention exposure

A dummy variable represented children’s exposure to the *responsive stimulation intervention* (*N =* 660) and a separate dummy variable was created to control for children’s exposure to the *enhanced nutrition intervention* (*N =* 626). To account for the factorial design of the PEDS trial [36], dummy variables for each intervention, along with an interaction term, were included in all regression models. The effects of the interventions on SDQ outcomes have been previously published [41], and thus we treat intervention exposure as control variables.

### Demographic characteristics

The following covariates were assessed by primary caregiver’s report: (a) *family wealth* at baseline using a single standardized factor score from a principal components analysis [42] of 44 items that reflect ownership of property, livestock, and household assets (e.g., television, bicycle, car), dwelling characteristics (e.g., access to water, sanitation facilities, type of flooring material), and number of bedrooms in the home ([43]; *M* = 0.00, *SD* = 0.99, *range* = -1.01–4.60); (b) *maternal education* measured the number of years the mother attended formal school (*M* = 2.19, *SD* = 3.69, range = 0–16); (c) total *number of children* in a household (*M* = 2.50, *SD* = 2.31, range = 0–11); and (d) *child’s gender* (1 = male).

## Results

### Data reduction

Daily and weekly briefings with assessors provided valuable information about how mothers perceived and responded to the SDQ questions. After reviewing this information, we learned that eight items were culturally inappropriate for preschoolers and had to remove them from future analyses.

- **Culturally unfamiliar item:** Assessors shared that the somatic symptoms described in item 3 “*often complains of headaches*, *stomach aches*, *or sickness*” were a culturally unfamiliar concept. Mothers had trouble differentiating between children who were frequently ill and children who reported somatic symptoms associated with anxiety.
- **Item not age-appropriate in Pakistan:** Assessors explained that the item 8 “*many worries*, *often seems worried”* was not age-appropriate for the Pakistani context, as many mothers expressed that four-year-old children are too young to be worried.
- **Items not considered to be behavioral problems:** There were two items (6 “*rather solitary*, *tends to play alone*” and 23 “*gets on better with adults than with other children*”) that were not considered to be behavioral problems in the context. Mothers expressed that many preschool-age children were not allowed to play outside of the home and so it is normative for them to play alone or spend time with adults in the home.
- **Items biased by probes:** Assessors reported that the items 21 “*thinks things out before acting*” and 25 “*sees tasks through to the end*, *good attention span*,” were likely biased by probes that ended up limiting, rather than aiding interpretation. For item 21, assessors asked mothers whether children thought before speaking in front of guests. Assessors reported that mothers responded very narrowly to the prompt and responses reflected how children reacted to guests in the home. For item 25, assessors asked mothers “if the child runs an errand, can they complete the errand without getting distracted along the way?” When debriefed, assessors reported that mothers often responded “yes” when their child was distractible because they missed the negative qualifier “*without* getting distracted.”
- **Developmentally inappropriate**: Items 18 “*often lies or cheats*” and 22 “*steals from home*, *school*, *or elsewhere*” were not found to be developmentally-appropriate for four-year-old children in our sample, similar to Ezpeleta and colleagues [44]. This is further corroborated by the fact that the items had very low variability. Seventy-four percent of parents reported that children did not “*lie or cheat*” and 92% of parents reported that children did not “*steal from home*, *school*, *or elsewhere*.”

### Preliminary confirmatory factor analysis with original subscales

Factor analyses were conducted using Mplus, Version 7.3 [45] and all analyses used weighted least squares means- and variance-adjusted (WLSMV) estimation, as recommended for ordered categorical variables and maximum likelihood estimation to account for missing data. All factor loadings are standardized. We report ordinal alpha reliability coefficients for the factors, because Cronbach’s alpha underestimates reliability for ordinal scales with few response categories [46]. Model fit was evaluated using Comparative Fit Index (CFI), Tucker Lewis Index (TLI), Root Mean Square Error of Approximation (RMSEA), chi-square test (χ^2^), and Weighted Root Mean Square Residual (WRMR). Values above 0.95 indicate good fit for the CFI and TLI. The cut-offs of 0.01, 0.05, and 0.08 have been used to indicate excellent, good, and mediocre fit, respectively, using the RMSEA [47]. The model chi-square value indicates good fit with a non-significant *p*-value. However, with samples larger than 400 chi-square statistics are almost always significant and are less useful indicators of model fit [48]. The WRMR is an experimental statistic and is currently not recommended [49], particularly with large sample sizes [50], and is only available for confirmatory factor analyses. Generally, smaller values are better with a recommended upper limit of 1.0.

To examine whether our data supported the original five-factor model [9], we conducted a confirmatory factor analysis using the original subscales with the eight problematic items removed (Table 2). Overall, the model had poor fit (CFI = 0.750; TLI = 0.688; RMSEA = 0.069; χ^2^(109) = 785.471, *p* < .001; WRMR = 2.050) and ordinal alphas for the subscales were low (Prosocial Behaviors α = .64, Peer Problems α = .51, Hyperactivity-Inattention α = .52, Emotional Problems α = .46, Conduct Problems α = .53).

**Table 2:** Factor loadings for confirmatory factor analysis of original five-factor solution with problematic items removed.

Factor and item			Loading	*SE*	*p*-value
Prosocial					
	1	Considerate	0.639	0.040	< .001
	4	Shares	0.462	0.039	< .001
	9	Helpful	0.553	0.040	< .001
	17	Kind to younger children	0.632	0.044	< .001
	20	Volunteers to help others	0.249	0.042	< .001
Peer problems					
	11	One good friend	0.564	0.043	< .001
	14	Liked by other children	0.714	0.041	< .001
	19	Picked on or bullied	-0.334	0.041	< .001
Hyperactivity/inattention					
	2	Restless, overactive	0.707	0.037	< .001
	10	Constantly fidgeting or squirming	0.666	0.037	< .001
	15	Easily distracted	0.363	0.043	< .001
Emotional problems					
	13	Unhappy, down heartened, tearful	0.836	0.112	< .001
	16	Nervous or clingy in new situations	0.288	0.051	< .001
	24	Many fears, easily stressed	0.194	0.048	< .001
Conduct problems					
	5	Often has temper tantrums	0.553	0.037	< .001
	7	Obedient, does what adults request	-0.471	0.036	< .001
	12	Fights with other children or bullies them	0.577	0.034	< .001

For model modification, we evaluated all pathways with modification indices above 10 and with high expected parameter change (EPC ≥ |0.40|) to judge if the proposed pathway made conceptual sense with the original subscales. We added the three suggested pathways that made conceptual sense and the fit improved slightly (CFI = 0.771; TLI = 0.707; RMSEA = 0.067; χ^2^(106) = 724.929, *p* < .001; WRMR = 1.945). Given the poor fit, even after the inclusion of modification indices, and low reliability of the factors, we decided to investigate the underlying factor structure using exploratory factor analysis.

### Exploratory factor analysis

We randomly selected half of the participants to examine the factor structure and reserved the second, independent half of participants to confirm these results. We conducted exploratory factor analysis (EFA) with 17 items (omitting those that were culturally-inappropriate) using an oblique quartimin rotation that allowed for correlations among factors. We tested solutions from one factor to five factors, given that the original scale had a five-factor structure [9] and that other researchers have found that structures with fewer factors fit the data better [51–53]. The number of factors to be extracted from the EFA was determined using parallel analysis, which is considered the most accurate method to decide the number of factors to retain [54,55]. Eigenvalues from our real data were compared to those produced by random data, and the three factors with eigenvalues greater than the random eigenvalues were retained.

The model fit for the three-factor solution from the exploratory factor analysis was better than the originally specified five-factor solution (CFI = 0.929; TLI = 0.890; RMSEA = 0.042; χ^2^(88) = 188.081, *p* < .001) and item loadings are presented in Table 3. To explore why the CFI and TLI were lower than expected for an exploratory analysis, we examined the null (or independence) model where all 17 of the SDQ items were assumed to have no correlation. We found that the RMSEA of the null model was 0.119, indicating that there are low correlations among the variables. It is not recommended to use incremental measures of fit (CFI and TLI) when the null model has an RMSEA of less than 0.158 [48]. This is because the TLI and CFI both depend on the average size of correlations between items in the data and are mechanically reduced when item correlations are low, so a model whose null model RMSEA is less than 0.158 and whose RMSEA is 0.05 must have a TLI and CFI of less than 0.90. With this knowledge, we decided to pursue a multi-faceted approach to making decisions about factor structure that included the evaluation of model fit statistics against standard thresholds, but also weighed in conceptual coherence of the items within each factor and the reliability of the items within each factor.

**Table 3:** Factor loadings for exploratory factor analysis of three-factor structure on a random half of the sample.

		Factor 1	Factor 2	Factor 3
1	Considerate		0.605	
2	Restless or overactive	0.822		
4	Shares		0.364	
5	Temper tantrums	0.574		
7	Obedient		0.440	
9	Helpful		0.616	
10	Fidgety	0.572		
11	Has good friend		0.624	
12	Fights	0.571		
13	Unhappy	0.415		0.319
14	Liked by children		0.578	
15	Distractible			
16	Nervous or clingy			0.581
17	Kind to younger children		0.566	
19	Bullied			
20	Volunteers to help		0.507	
24	Many fears, easily stressed			0.634
Factor Intercorrelations				
	Factor 1	___		
	Factor 2	-0.195	___	
	Factor 3	0.021	-0.151	___

Rotated quartimin four-factor solution using the WLSMV estimator to account for ordinal data. Coefficients less than +/- 0.3 were omitted. N/A = not applicable. All loadings were significant at the *p* < .05 level.

We then examined the conceptual coherence of the items in the factors from the exploratory factor analysis. Factor 1 (externalizing behavior problems) had five items that represented externalizing problems such as “*temper tantrums*,” “*fidgeting*,” and “*unhappy*”. Factor 2 (prosocial behaviors) had eight items that represented children’s positive behaviors with peers (“*liked by children*,” “*shares*”) and adults (“*obedient*,” “*volunteers to help others*”). Factor 3 (anxiety problems) had three items that represented children’s internalizing behavioral problems (*“unhappy*,*” “nervous or clingy*, *easily loses confidence*,*” “many fears*, *easily stressed”*). Item 13, “*unhappy*,” cross-loaded on both Factor 1 (loading = 0.415) and Factor 3 (loading = 0.319). Two items 15 “*easily distracted*, *concentration wanders*” and 19 “*picked on or bullied by other children*” did not load on any factors higher than 0.3, so we removed them and were left with fifteen items.

### Final confirmatory factor analysis

Finally, we ran a confirmatory factor analysis using the fifteen items that significantly loaded on the three factors in the EFA using the second random half of participants. Shown in Table 4, the confirmatory factor analysis demonstrated good fit as indexed by the RMSEA (RMSEA = .047) but poor fit as indexed by the CFI and TLI (CFI = 0.865, TLI = 0.835 χ^2^(86) = 231.174, *p* < .001; WRMR = 1.239). As expected, Factor 1 (externalizing behavior problems) was negatively associated with Factor 2 (prosocial behaviors; β = -0.378; *p* < .001). Factor 3 (anxiety problems) was not significantly correlated with any other factors.

**Table 4:** Factor loadings for confirmatory factor analysis of three-factor solution from the EFA model on the other random half of the sample.

Factor and item			Loading	*SE*	*p*-value
Externalizing behavior problems					
	2	Restless or overactive	0.616	0.048	< .001
	5	Temper tantrums	0.564	0.051	< .001
	10	Fidgety	0.619	0.048	< .001
	12	Fights	0.634	0.047	< .001
	13	Unhappy	0.443	0.058	< .001
Prosocial behaviors					
	1	Considerate	0.558	0.055	< .001
	4	Shares	0.427	0.053	< .001
	7	Obedient	0.473	0.051	< .001
	9	Helpful	0.469	0.056	< .001
	11	Has good friend	0.485	0.058	< .001
	14	Liked by children	0.724	0.049	< .001
	17	Kind to younger children	0.654	0.057	< .001
	20	Volunteers to help	0.193	0.057	.001
Anxiety problems					
	13	Unhappy	0.087	0.069	.205
	16	Nervous or clingy	1.363	0.849	.109
	24	Many fears, easily stressed	0.256	0.116	.123

WLSMV estimator to account for ordinal data. Factor intercorrelations: Externalizing Behavior Problems & Prosocial Behaviors: *β* = -0.378, *p* < .001; Externalizing Behavior Problems & Anxiety Problems: β = 0.035, *p* = .497; Prosocial Behaviors & Anxiety Problems: *β* = -0.158, *p* = .151.

### Social-emotional behavior composite measures

We created two composite measures of *externalizing behavior problems* and *prosocial behaviors* by averaging the items in Factor 1 and Factor 2, respectively, to allow for replicability in future studies. These factors were conceptually coherent, had acceptable reliability (externalizing behavior problems α = .72, prosocial behaviors α = .74), and had items that loaded significantly in expected directions. Finally, we did not create a composite measure of anxiety problems because the items did not load significantly onto the factor in the final CFA (item 13: *β* = 0.087, *p* = .205, item 16: *β* = 1.363, *p* = .109, item 24: *β* = 0.256, *p* = .123), the factor was not correlated with the other two factors, and the items in the factor had very low reliability (α = .41). Further, model fit was slightly improved when we conducted a CFA with only Factors 1 and 2 (RMSEA = .059; CFI = 0.858, TLI = 0.827; χ^2^(64) = 208.465, *p* < .001; WRMR = 1.336).

### Descriptive statistics and bivariate associations

Table 5 shows bivariate associations between all study variables. Bivariate associations showed that male children and those who experienced food insecurity had higher externalizing behavior problems and fewer prosocial behaviors. Children with more siblings had fewer externalizing behavior problems. Mothers with a greater number of depressive symptoms reported higher rates of child externalizing behavior problems, but maternal depressive symptoms were not associated with prosocial behaviors. Wealth, maternal education, and the quality of the home environment were positively correlated with prosocial behaviors, but uncorrelated with externalizing behavior problems. Finally, measures of early physical, cognitive/language, and social-emotional skills were positively associated with prosocial behaviors, but uncorrelated with externalizing behavior problems.

**Table 5:** Bivariate correlations for social-emotional behaviors and family- and child-level correlates.

	1	2	3	4	5	6	7	8	9	10	11	12	13
1. Ext BPs	___												
2. Prosocial behav	**-.19**	___											
3. Male child	**.17**	**-.08**	___										
4. Mat educ	.00	**.11**	.02	___									
5. Family wealth	-.04	**.11**	-.01	**.37**	___								
6. Num siblings	**-.09**	.04	.00	**-.16**	**-.12**	___							
7. RS	.02	**.19**	.03	-.01	.04	.02	___						
8. EN	-.01	**-.08**	-.05	**.09**	-.01	-.03	-.01	___					
9. Mat dep (12m)	**.07**	-.02	.01	**-.10**	**-.12**	**.15**	.02	**-.09**	___				
10. Home (18m)	.02	**.24**	-.03	**.28**	**.30**	**-.16**	**.39**	**.10**	**-.10**	___			
11. Food in (24m)	**.08**	**-.06**	.04	**-.22**	**-.25**	**.12**	**-.07**	**-.14**	**.21**	**-.24**	___		
12. Height (24m)	.01	**.10**	-.02	**.23**	**.27**	**-.10**	-.04	.03	**-.09**	**.23**	**-.17**	___	
13. BSID soc/emo	.02	**.15**	.01	**.14**	**.10**	**-.08**	-.02	.09	**-.11**	**.23**	**-.14**	**.22**	___
14. BSID cog/lang	.04	**.16**	.01	**.21**	**.25**	**-.12**	**.28**	**.09**	**-.06**	**.42**	**-.22**	**.35**	**.36**

Bolded values are significant at *p* < .05. Ext BPs = externalizing behavior problems; mat educ = maternal education; num = number of; RS = responsive stimulation intervention; EN = enhanced nutrition intervention; mat dep = maternal depressive symptoms; home = home environment quality; food in = food insecurity; BSID = BSID-III; soc/emo = social-emotional; cog/lan = cognitive/language

### Regression analyses: Family- and child-level correlates of social-emotional behaviors

To explore family- and child-level correlates of children’s externalizing behavior problems and prosocial behaviors, we conducted linear regressions. Missing data was addressed using multiple imputation with chained equations (MI) using 20 datasets. As shown in Table 1, rates of missing data were negligible, ranging from 0.00% to 2.69%. The models included demographic characteristics at baseline (gender, number of siblings, maternal education, family wealth), intervention exposure (responsive stimulation, enhanced nutrition, and an interaction between both interventions), measures of children’s family experiences (maternal depressive symptoms, quality of the home environment, food insecurity) and child-level developmental competencies at two years (social-emotional skills, cognitive/language skills, height-for-age).

As shown in Table 6, male children had significantly more externalizing behavior problems (*β* = 0.344, *p* < .001, *η*^2^ = 0.029) and children with siblings had fewer externalizing behavior problems (*β* = -0.109, *p* < .001, *η*^2^ = 0.010). Measures of maternal education and wealth were not associated with children’s externalizing behavior problems. As published in Yousafzai et al. 2016, an interaction between the responsive stimulation and enhanced nutrition interventions emerged (*β* = -0.236, *p* = .046), such that the effect of the responsive stimulation intervention on children’s externalizing behavior problems was weaker for children who also received the enhanced nutrition intervention. Food insecurity and maternal depressive symptoms were associated with higher externalizing behavior problems (*β* = 0.187, *p* = .007, *η*^2^ = 0.006; *β* = 0.056, *p* = .014, *η*^2^ = 0.003). Measures of children’s social-emotional skills, cognitive/language skills, and height-for-age at two years were not linked to children’s externalizing behavior problems at four years.

**Table 6:** Regression models predicting social-emotional behaviors from family- and child-level correlates.

		Externalizing Behavior Problems		Prosocial Behaviors	
		*β*	*(SE)*	*β*	*(SE)*
Baseline demographics					
	Male child	0.344	(0.048)***	-0.172	(0.053)**
	Number of siblings	-0.110	(0.029)***	0.086	(0.026)**
	Maternal education	0.006	(0.029)	0.055	(0.026)*
	Family wealth	-0.043	(0.030)	0.029	(0.030)
Exposure to interventions					
	RS	0.137	(0.087)	0.006	(0.075)
	EN	0.133	(0.090)	-0.471	(0.091)***
	RS x EN	-0.236	(0.116)*	0.467	(0.113)***
Family experience					
	Food insecurity (24 m)	0.187	(0.068)**	-0.008	(0.061)
	Maternal dep symptoms (12 m)	0.056	(0.022)*	0.006	(0.032)
	Home environment (18 m)	0.006	(0.034)	0.158	(0.038)***
Early development					
	BSID social-emotional (24 m)	0.006	(0.026)	0.114	(0.025)***
	BSID cognition/language (24 m)	0.032	(0.033)	0.036	(0.034)
	Height-for-age (24 m)	0.025	(0.027)	0.019	(0.029)
Constant		-0.322	(0.069)***	0.205	(0.062)**
*R*^*2*^		.058		.125	
*N*		1302		1302	

* *p* < .05

** *p* < .01

*** *p* < .001. RS = responsive stimulation intervention; EN = enhanced nutrition intervention; dep = depressive, m = months

Biological, socioeconomic, and family factors were associated with children’s prosocial behaviors. Male children were reported to have fewer prosocial behaviors (*β* = -0.172, *p* = .002, *η*^2^ = 0.008) and children with more siblings had higher prosocial behaviors (*β* = 0.086, *p* = .002 , *η*^2^ = 0.007). Maternal education was linked to higher prosocial behaviors (*β* = 0.055, *p* = .037, *η*^2^ = 0.003), but wealth was not linked to prosocial behaviors. The main effect of the enhanced nutrition intervention was qualified by a positive interactive effect between the two interventions (*β* = 0.467, *p* < .001), such that the positive effects of the responsive stimulation intervention were magnified by the enhanced nutrition intervention (see [41] for more details). Home environment quality, but not measures of food insecurity and maternal depressive symptoms, was positively associated with prosocial behaviors (*β* = 0.158, *p* < .001, *η*^2^ = 0.015). Finally, children’s social-emotional skills at two years were associated with prosocial behaviors at four years (*β* = 0.114, *p* < .001, *η*^2^ = 0.010), but children’s cognitive/language skills and height-for-age at two years were not associated with their prosocial behaviors.

## Discussion

There is growing interest in how to assess young children’s social-emotional behaviors in highly-disadvantaged contexts. For this study, an interdisciplinary team of researchers and community members adapted a commonly-used parent-report of children’s social-emotional behaviors for use in rural Pakistan. Using a birth cohort of four-year-old children, and controlling for their exposure to early interventions, we found that the original five-factor structure and reliability of the original SDQ subscales were not acceptable. Factor analyses revealed that three factors better fit the data and two composite measures of children’s externalizing behavior problems and prosocial behaviors had adequate reliability. Finally, we found evidence for the validity of our measures. Early exposure to maternal depression and food insecurity were linked to higher rates of later externalizing behavior problems. Maternal education, earlier home environment quality, and a measure of social-emotional competencies at two years were linked to prosocial behaviors at four years. Our results indicate that externalizing behavior problems and prosocial behaviors can be reliably assessed via parent report in LMIC with high levels of illiteracy and unfamiliarity with Likert-scale questionnaires.

### Structure of the strengths and difficulties questionnaire

Confirmatory factor analyses demonstrated that the original five-factor SDQ structure did not fit our data. Items within the five subscales showed low or unexpected correlations. A recent study in Pakistan similarly showed that adolescents’ self-reported SDQ scores did not fit the original five-factor structure [56]. While the original subscales were unreliable, we leveraged item-level responses to create conceptually- and empirically-coherent measures of children’s social-emotional behaviors. When we explored the factor structure in our sample, three factors emerged: externalizing behavior problems, prosocial behaviors, and anxiety problems. Only the externalizing behavior problems and prosocial behaviors composites had adequate reliability for use in regression analyses. These factors had a low correlation, suggesting that they captured unique aspects of children’s social-emotional behaviors.

In this sample, we were not able to reliably assess other dimensions of children’s social-emotional behaviors. We learned several lessons through evaluation of the qualitative data provided by assessors. First, mothers were unfamiliar with concepts on the original Emotional Problems subscale. For instance, mothers had trouble differentiating between somatic symptoms associated with anxiety and children’s physical illnesses. Second, assessors reported that several constructs were not culturally appropriate for preschool-age children in the Pakistani context, including worrying and solitary behavior. Third, parents struggled to understand items about attentional issues and probes did not clarify these items, as they included multiple clauses and qualifying statements. Finally, our results suggest that items assessing deviant behaviors such as lying, cheating, and stealing may not be developmentally-appropriate for preschoolers. We suggest following Ezpeleta and colleagues [44] by substituting them for measures of oppositional behaviors in early childhood samples. Overall, we hope that these lessons can guide the development and adaptation of other instruments to measure social-emotional behaviors in LMIC, as they provide general categories of issues that may arise.

Despite the challenges associated with use of parent report measures in LMIC, we reliably assessed certain aspects of children’s social-emotional behaviors. When adapting measures for new contexts, we recommend that researchers engage in extensive piloting processes involving local members of the community in the feedback and revision processes. This will help identify items that are not appropriate for the context, items that need to be reworded, and items that would benefit from additional examples or probes. The qualitative data provided by assessors and community members was crucial for adapting the SDQ to make it accessible to mothers in this context and to interpret our findings. When questionnaires are unreliable, we highlight the opportunity to use item-level data to create meaningful measures of children’s skills.

### Correlates of social-emotional behaviors in rural Pakistan

Analysis of child- and family-level longitudinal predictors revealed that girls and children with more siblings showed higher prosocial skills and lower externalizing behavior problems, compared to boys and those with fewer siblings. Consistent with our findings, studies with Pakistani primary-school students have found that boys have higher rates of Conduct Problems and Hyperactivity-Inattention on the SDQ, compared to girls [14,15,28]. However, these studies did not find that boys exhibited fewer prosocial behaviors compared to girls [15,28]. The only study that examined whether children with more siblings have better social-emotional behaviors in Pakistan, did not find an association between siblings and the original SDQ subscales in a primary school sample [28]. This may be because once children enter school, their experiences with peers become more salient predictors of their social-emotional development. Less than ten percent of preschoolers in Pakistan are enrolled in early education programs [33], and thus siblings may provide unique opportunities for young children to practice and improve their social-emotional skills in early childhood, similar to findings in the United States [34].

### Unique correlates of children’s externalizing behavior problems

Earlier household food insecurity and maternal depressive symptoms emerged as unique predictors of children’s externalizing behavior problems, whereas indices of socioeconomic status were unrelated to children’s externalizing behavior problems at four years. A recent study of Pakistani seven-year-olds similarly did not find socioeconomic differences on the SDQ Conduct Problems and Hyperactivity-Inattention subscales which most closely align with our measure of externalizing behavior problems [28]. Parents who experienced household food insecurity when the child was two years reported higher levels of child externalizing behavior problems at four years. In the United States, household food insecurity has been recently linked to children’s social-emotional development in early childhood [57]. Most research in LMIC has focused on the negative effects of food insecurity on children’s health outcomes [58,59], and one study demonstrated that food insecurity is linked to worse educational outcomes in middle childhood [60]. We are the first to report an association between early food insecurity and preschoolers’ behavior problems in LMIC. Interestingly, including child height-for-age at two years did not reduce the effect of food insecurity on children’s externalizing behavior problems. This suggests that food insecurity is not influencing children’s externalizing behavior problems via undernutrition, but possibly through increased psychological stress in the household. There is a larger evidence base supporting the negative effects of maternal depressive symptoms on children’s social-emotional development in LMIC [28,30,31]. A recent study in Pakistan, found that maternal depression during the prenatal period and when the child was seven years, were each linked to increased total behavioral difficulties on the SDQ [28].

We conceptualized both maternal depressive symptoms and food insecurity as indicators of maternal psychological distress. In LMIC, food insecurity has been linked to adult depressive symptoms, because of the psychological stress associated with inconsistent access to food [61]. We posit that emotionally-distressed mothers have less positive interactions with their children, which are important for building early regulatory competencies [62]. A study in Uganda showed that maternal depressive symptoms affected children’s externalizing behavior problems through increases in harsh and inconsistent parenting [31], suggesting that the mechanisms linking parental distress to child behavior may be similar in both low- and high-income contexts. Alternatively, these findings may be driven by maternal reporting bias. Mothers who are depressed, anxious, or stressed are more likely to report higher levels of externalizing behavior problems than teachers, trained clinicians, and children themselves [63,64]. However, it is important to note that there were two to three intervening years between maternal report of distress (depressive symptoms and food insecurity) and children’s behavioral problems, so this is less probable.

It is notable that a measure of children’s social-emotional skills at two years was not associated with children’s externalizing behavior problems at four years. This is likely because the BSID-III social-emotional scale did not measure precursors of children’s early externalizing behavior problems. The social-emotional scale is focused on measuring early communication of needs and play behaviors and does not measure typical predictors of externalizing behaviors such as negative emotionality, fussiness, aggression, and impulsivity [65,66]. It is also plausible that the externalizing behavior problems composite is not capturing the construct intended. Our externalizing behavior problems composite may instead be picking up on mothers who are distressed in their role as a parent, and therefore rate their children as having more behavioral issues on the SDQ, instead of children with higher behavioral difficulties. Given that externalizing behavior problems pose significant long-term academic and social difficulties [1,67], it will be important to measure early indicators of behavioral regulation difficulties in Pakistan.

### Unique correlates of children’s prosocial behaviors

Maternal education, home environment quality, and social-emotional skills at two years were unique correlates of prosocial behaviors. Mothers who attended more years of school rated their children as having better prosocial skills. This aligns with one study demonstrating that Pakistani seven-year-olds from more socioeconomically-advantaged contexts have higher prosocial skills on the SDQ [29]. Children in higher-quality home environments, measured by the total score on the HOME, also displayed more prosocial behaviors. Across many LMIC, the quality of the home environment has been linked to composite measures of children’s early development, including pre-academic skills, physical development, social-emotional skills, and approaches to learning [33,68]. While the HOME captures opportunities for stimulation in the home, many items reflect family socioeconomic status (e.g. toys and resources in the home, safe environment]. It is possible that children from more advantaged homes have better prosocial skills due to socioeconomic differences in parental socialization, parental expectations for behavior, and opportunities to interact with other children outside of the home.

In contrast with externalizing behavior problems, measures of food insecurity and maternal depressive symptoms were not associated with children’s prosocial behaviors. Maselko and colleagues similarly found that maternal depression was not associated with Peer Problems or Prosocial Skills in Pakistani seven-year-olds [29]. Most other studies in LMIC have used a composite measure of total difficulties on the SDQ [31,69], making it difficult to disentangle whether maternal depression is differentially linked to specific social-emotional constructs. It is possible that maternal distress is not predictive of mothers’ ratings of prosocial behaviors because the development of prosocial skills requires the presence of positive interactions with children and adults, rather than just the lack of maternal distress.

Finally, the BSID-III social-emotional scale at two years was highly predictive of prosocial behaviors at four years. The BSID-III has been widely-used to capture toddlers’ play behaviors, communication of needs and feelings, and use of emotional signals to solve problems (e.g. [67,70]), which link conceptually to the emotional awareness and communication skills that support prosocial behaviors. This finding provides support for the construct validity of our prosocial behaviors composite. To our knowledge, ours is the first study in LMIC demonstrating developmental continuity of early social-emotional behaviors. This has important implications for the development of early interventions and family supports that promote children’s communication skills and provide them with opportunities to engage in play during the first two years.

### Limitations and future directions

While translating questionnaires enables cross-cultural comparison, the measures may miss important culturally-relevant skills. For example, in the Pakistani context, “respect for elders” would be viewed as a salient indicator of prosocial skills and “challenging others” would be viewed as a behavioral problem. These skills are not captured in our current subscales and reflect how group goals emphasized by collectivistic cultures are not evaluated in the SDQ. In future work, it will be crucial to create measures of social-emotional behaviors that capture children’s adaptive development in diverse settings. Second, due to time and resource constraints, we were only able to administer one measure of children’s social-emotional behaviors at the 4-year assessment. Direct assessments and observations could provide complementary information to better understand how preschoolers express social-emotional behaviors in LMIC. This would also eliminate issues of same-reporter bias when exploring links between mother-reported measures (such as maternal depression) and social-emotional behaviors.

Third, all of the items that loaded onto the externalizing behavior problems factor were worded negatively (i.e. fidgety, fights) and all of the items that loaded on the prosocial behaviors factor were worded positively (i.e. considerate, shares). It is possible that these items grouped together because they represented undesirable and desirable behaviors in the Pakistani context, rather than by the content of the items. However, there is evidence that mothers were able to distinguish between externalizing- and internalizing-type items, given that two distinct factors arose (externalizing behavior problems and anxiety problems). Mothers had more trouble differentiating between prosocial behaviors (e.g. shares, considerate) and the quality of children’s peer relationships (e.g. has a good friend, liked by other children) as items from both of these original subscales were grouped together in the prosocial behaviors composite. Future research should explore whether the wording (positive or negative) of questions about children’s behaviors influences how disadvantaged mothers in LMIC respond to them.

Fourth, longitudinal research is needed to explore the predictive validity of social-emotional behaviors in early childhood for children’s academic performance and relationships with peers. There is little research examining how early social-emotional behaviors contribute to children’s developmental trajectories in LMIC. Finally, despite including a robust set of child- and family-level predictors, including a prior measure of social-emotional skills at two years, we only explained a small amount of the variance in children’s social-emotional behaviors. More work is needed to learn about what shapes the development of social-emotional behaviors in global contexts.

### Conclusion

Our study highlights the importance of examining item functioning and the reliability of questionnaires *after* administration, which may be especially useful in large-scale trials where time for adaptation and testing is often limited. Leveraging item-level data, we created two reliable measures of children’s social-emotional behaviors. Our findings suggest that certain items, particularly from the original Prosocial Skills and Conduct Problems SDQ subscales, may be most accessible to mothers of preschoolers in LMIC. In future studies, researchers may want to focus on these items as developmentally-appropriate and easily-understood aspects of young children’s social-emotional behaviors.

Finally, we found that many well-established predictors of social-emotional development also applied in rural Pakistan. The predictors differed depending on the type of social-emotional behavior, suggesting that early experiences of parental emotional distress may be linked to externalizing behavior problems whereas socioeconomic status may be more predictive of prosocial development. We hope that this work is extended to other LMIC, to shed light on which early experiences universally promote social-emotional development and which may be unique to different contexts.

## Supporting information

S1 Appendix Adaptation of the strengths and difficulties questionnaire. (DOCX)

S2 Appendix Final item wording and bivariate correlations for all SDQ items. (DOCX)

S3 Appendix Final items for measures in English and Sindhi. (DOCX)

S1 File Data for all analyses in study. (CSV)
