# Supplementary material for: Measuring and understanding social-emotional behaviors in preschoolers from rural Pakistan
Source: PLoS One. 2018 Nov 27;13(11):e0207807. doi: 10.1371/journal.pone.0207807 (PMC6258542; doi:10.1371/journal.pone.0207807)
Supplement: S1 Appendix — (DOCX) [file pone.0207807.s001.docx]

S1 Appendix

Adaptation of the Strengths and Difficulties Questionnaire

This appendix describes the procedures involved in adapting the Strengths and Difficulties Questionnaire (SDQ) for use in the Sindh province of Pakistan.

**Expert Discussion**

First, a team of child development researchers and women from the local community met to discuss the SDQ items. They decided to make minor modifications to six items shown in Table A. They also chose to administer the SDQ in an interview format because of low literacy levels in the data collection region.

Table A

| Item # | English | Modification |
| --- | --- | --- |
| 1 | Considerate of other people’s feelings | Add probe for clarification (e.g. if you are upset, does CHILD notice? Does CHILD try not do or say things that can hurt others’ feelings?) |
| 10 | Constantly fidgeting or squirming | Need clarification to explain to mother (e.g. child cannot sit still, or is constantly fiddling with hands) |
| 13 | Often unhappy, down hearted or tearful | Simpler words are needed for the population than the less familiar term used in the available Urdu version  Suitable words for down hearted in Urdu discussed: *dil ochat hona, dil barastha, past hosla rehna, buhji se tabiat.* |
| 15 | Easily distracted, concentration wanders | Add probe for clarification to mother (e.g. If you speaking to CHILD does he/she listen to what you are saying, or does he lose interest?) |
| 21 | Thinks things out before acting | Add probe for clarification (e.g. Does CHILD plan out a task before completing it?)  Emphasize behaviors or actions |
| 25 | Sees tasks through to the end, good attention span | Add probe for clarification to mother (e.g. If you ask CHILD to run an errand like go the market stall, will he/she be able to complete the errand without getting distracted?) |

**Create Translated Forms**

After the initial modifications listed above were made in Urdu, the questionnaire was translated to Sindhi, the local language. From Sindhi, the SDQ was independently back-translated into English to ensure that items were similar to the original SDQ questionnaire, written in English. Following a cycle of discussions with local community members and testing with a small group of mothers, the translations were further refined to ensure the items were properly translated into Sindhi.

**First Pilot Testing**

The measure was then pilot tested on a sample of 7 mothers of preschoolers drawn from the study area (but with children who were not in the study). They noted that it took 10 to 15 minutes to administer the SDQ in the interview format. Six items needed minor modifications (listed below in Table B). After these modifications were made, the item was finalized in Sindhi and back-translated to English.

Table B

| Item # | English | Modification |
| --- | --- | --- |
| 2 | Restless, overactive, cannot stay still for long | Need to emphasize that overactive is more than normal activity |
| 5 | Often has temper tantrums or hot tempers | Need to have one more Sindhi word for ‘tantrums’ |
| 8 | Many worries, often seems worried | Need examples to clarify children’s ‘worries’ |
| 12 | Often fights with other children or bullies them | Need one more Sindhi word for ‘threat’ |
| 15 | Easily distracted, attention wanders | Change Sindhi word for ‘attention wanders,’ examples needed to clarify |
| 20 | Often volunteers to help others (parents, teachers, other children) | Change Sindhi word for ‘helps’ |

**Second Pilot Testing**

The measure was then pilot tested on a sample of 25 mothers of preschoolers drawn from the study area (but with children who were not in the study). The assessors reported confusion about how to conduct inter-rater reliability. The team of child development experts and assessor trainers clarified that two assessors should score the *same* administration of the SDQ (rather than two separate administrations).

First, descriptives and distributions of each of the original subscales (emotional symptoms, conduct problems, hyperactivity, peer problems, and prosocial skills) was analyzed. As shown in Table C, distributions for each of the subscales was found to be normal for emotional symptoms, conduct problems, hyperactivity, and peer problems. The prosocial scale had a slight negative skew, suggesting that mothers may have reported more positively on children’s prosocial skills.

Table C

| Scale | *M* | SD | Range | Skew |
| --- | --- | --- | --- | --- |
| Prosocial behaviors | 7.08 | 2.38 | 1 – 10 | -1.03 |
| Peer problems | 3.48 | 1.36 | 1 – 7 | 0.40 |
| Hyperactivity-inattention | 3.64 | 1.55 | 1 – 6 | -0.34 |
| Emotional problems | 3.84 | 2.37 | 0 – 9 | 0.19 |
| Conduct problems | 2.84 | 2.21 | 0 – 8 | 0.73 |

Second, response variation for each item was examined. Only one item (22 “*steals* *from home, school, or elsewhere*”) showed little variation across the three response options, which is not surprising given that the children were preschoolers.

Table D

| Item # | Description | Not true | Somewhat true | Certainly true |
| --- | --- | --- | --- | --- |
| 1 | Considerate | 8 % | 28 % | 64 % |
| 2 | Restless | 40 % | 28 % | 32 % |
| 3 | Somatic sickness | 40 % | 36 % | 24 % |
| 4 | Shares | 20 % | 32 % | 48 % |
| 5 | Temper tantrums | 16 % | 48 % | 36 % |
| 6 | Solitary, plays alone | 44 % | 40 % | 16 % |
| 7 | Obedient | 48 % | 44 % | 8 % |
| 8 | Many worries | 72 % | 20 % | 8 % |
| 9 | Helpful | 12 % | 36 % | 52 % |
| 10 | Fidgeting | 56 % | 16 % | 28 % |
| 11 | One good friend | 60 % | 28 % | 12 % |
| 12 | Fights / bullies | 52 % | 32 % | 16 % |
| 13 | Unhappy | 44 % | 44 % | 12 % |
| 14 | Liked by other children | 60 % | 36 % | 4 % |
| 15 | Distractible | 36 % | 48 % | 16 % |
| 16 | Nervous / clingy | 36 % | 28 % | 36 % |
| 17 | Kind to younger children | 0 % | 32 % | 68 % |
| 18 | Often lies or cheats | 76 % | 8 % | 16 % |
| 19 | Picked on / bullied | 28 % | 56 % | 16 % |
| 20 | Volunteers | 24 % | 40 % | 36 % |
| 21 | Thinks before acting | 28 % | 48 % | 24 % |
| 22 | Steals | 92 % | 8 % | 0 % |
| 23 | Better with adults | 36 % | 40 % | 24 % |
| 24 | Many fears, easily stressed | 32 % | 36 % | 32 % |
| 25 | Good attention span | 80 % | 16 % | 4 % |

Third, they confirmed that there were no significant gender differences on the items (see Table E). Only item 5(“*temper tantrums*”) showed significant gender differences such that boys were reported to throw more temper tantrums than girls.

Finally, test-retest and inter-rater reliability were examined. Both test-retest (*r= 0*.94 – 1.00) and inter-rater reliability (*r = 1*.00) showed high Pearson correlation coefficients, supporting acceptable reliability across testing periods and raters.

Table E

|  |  | *Girls* | |  | *Boys* | | *t* | *p*-value |
| --- | --- | --- | --- | --- | --- | --- | --- | --- |
| Item # | Description | *M* | *SD* |  | *M* | *SD* |  |  |
| 1 | Considerate | 1.78 | 0.44 |  | 1.44 | 0.73 | 1.27 | .216 |
| 2 | Restless | 1.00 | 0.87 |  | 0.88 | 0.89 | 0.34 | .736 |
| 3 | Somatic sickness | 0.67 | 0.71 |  | 0.94 | 0.85 | 0.43 | .786 |
| 4 | Shares | 1.78 | 0.44 |  | 1.00 | 0.82 | -0.81 | .428 |
| 5 | Temper tantrums | 1.00 | 0.87 |  | 1.31 | 0.60 | 2.63 | .015 |
| 6 | Solitary, plays alone | 0.56 | 0.73 |  | 0.81 | 0.75 | -0.83 | .414 |
| 7 | Obedient | 0.56 | 0.73 |  | 0.63 | 0.62 | -0.25 | .802 |
| 8 | Many worries | 0.33 | 0.71 |  | 0.38 | 0.62 | -0.15 | .879 |
| 9 | Helpful | 1.56 | 0.53 |  | 1.31 | 0.79 | 0.82 | .421 |
| 10 | Fidgeting | 0.56 | 0.88 |  | 0.81 | 0.91 | -0.68 | .500 |
| 11 | One good friend | 0.33 | 0.50 |  | 0.63 | 0.81 | -0.98 | .338 |
| 12 | Fights / bullies | 0.78 | 0.83 |  | 0.56 | 0.73 | 0.67 | .507 |
| 13 | Unhappy | 0.56 | 0.73 |  | 0.75 | 0.68 | -0.67 | .511 |
| 14 | Liked by other children | 0.22 | 0.44 |  | 0.56 | 0.63 | -1.43 | .166 |
| 15 | Distractible | 0.89 | 0.78 |  | 0.75 | 0.68 | 0.46 | .647 |
| 16 | Nervous / clingy | 1.33 | 0.87 |  | 0.81 | 0.83 | 1.48 | .153 |
| 17 | Kind to younger children | 1.56 | 0.53 |  | 1.75 | 0.45 | -0.98 | .338 |
| 18 | Often lies or cheats | 0.33 | 0.71 |  | 0.44 | 0.81 | -0.32 | .751 |
| 19 | Picked on / bullied | 0.78 | 0.67 |  | 0.94 | 0.68 | -0.57 | .576 |
| 20 | Volunteers | 1.44 | 0.73 |  | 0.94 | 0.77 | 1.61 | .121 |
| 21 | Thinks before acting | 1.22 | 0.67 |  | 0.81 | 0.75 | 1.36 | .187 |
| 22 | Steals | 0.00 | 0.00 |  | 0.13 | 0.34 | -1.09 | .288 |
| 23 | Better with adults | 1.11 | 0.78 |  | 0.75 | 0.77 | 1.12 | .276 |
| 24 | Many fears, easily stressed | 1.11 | 0.93 |  | 0.94 | 0.77 | 0.50 | .620 |
| 25 | Good attention span | 0.44 | 0.73 |  | 0.13 | 0.34 | 1.50 | .146 |
