# Supplementary material for: Measuring and understanding social-emotional behaviors in preschoolers from rural Pakistan
Source: PLoS One. 2018 Nov 27;13(11):e0207807. doi: 10.1371/journal.pone.0207807 (PMC6258542; doi:10.1371/journal.pone.0207807)
Supplement: S2 Appendix — (DOCX) [file pone.0207807.s002.docx]

S2 Appendix

Final Item Wording and Bivariate Correlations for all SDQ Items

Table A

*Final Items and Probes/Examples from the Strengths and Difficulties Questionnaire in English*

| Item # | Description | Original Scale |
| --- | --- | --- |
| 1 | Considerate of other people’s feelings  *E.g. If you are upset, does CHILD notice? Does CHILD try not to do or say things that can hurt others’ feelings?* | Prosocial |
| 2 | Restless, overactive, cannot stay still for long | Hyperactivity |
| 3 | Often complains of headaches, stomach aches, or sickness | Emotional Problems |
| 4 | Shares readily with other children  *E.g. treats, toys, pencils, etc.* | Prosocial |
| 5 | Often has temper tantrums or hot tempers | Conduct Problems |
| 6 | Rather solitary, tends to play alone | Peer Problems |
| 7 | Generally obedient, usually does what adults request | Conduct Problems |
| 8 | Many worries, often seems worried | Emotional Problems |
| 9 | Helpful if someone is hurt, upset, or feeling ill | Prosocial |
| 10 | Constantly fidgeting or squirming  *E.g. CHILD cannot sit still or is constantly fiddling with hands.* | Hyperactivity |
| 11 | Has at least one good friend | Peer Problems |
| 12 | Often fights with other children or bullies them | Conduct Problems |
| 13 | Often unhappy, down hearted or tearful | Emotional Problems |
| 14 | Generally liked by other children | Peer Problems |
| 15 | Easily distracted, concentration wanders  *E.g. If you are speaking to CHILD does s/he listen to what you are saying or does s/he lose interest?* | Hyperactivity |
| 16 | Nervous or clingy in new situations, easily loses confidence | Emotional Problems |
| 17 | Kind to younger children | Prosocial |
| 18 | Often lies or cheats | Conduct Problems |
| 19 | Picked on or bullied by other children | Peer Problems |
| 20 | Often volunteers to help others  *E.g. Parents, teachers, or other children* | Prosocial |
| 21 | Thinks things out before acting  *E.g. Does CHILD think before speaking out in front of guests?* | Hyperactivity |
| 22 | Steals from home, school, or elsewhere | Conduct Problems |
| 23 | Gets on better with adults than other children | Peer Problems |
| 24 | Many fears, easily stressed | Emotional Problems |
| 25 | Sees tasks through to the end, good attention span  *E.g. If you ask CHILD to run an errand like go to the market stall, will s/he be able to complete the errand without getting distracted along the way?* | Hyperactivity |

Table B

*Final Items from the Strengths and Difficulties Questionnaire in Sindhi*

| نمبر | سوال |
| --- | --- |
|  |  |
|  | ٻين ماڻهن جي احساسن جو خيال رکڻ وارو آهي.  (مثال ماءُ کي پريشان ڏسي ته ان کان اچي پڇي) |
|  | بي آرام، حد کان وڌيڪ پهڙتيلو، هڪ جاء تي گهڻي ديرتائين رڪجي نه ٿو سگهي. |
|  | گهڻو ڪري مٿي ۾ سور، پٽ ۾ سور يا دل خراب ۽ الٽي جي شڪايت ڪندو آهي. |
|  | ٻين ٻارن سان شيون خوشي سان ورهائي کيڏندو آهي،  شيون ڏئي ٿو. (رانديڪا يا پينسلون وغيره). |
|  | گهڻو ڪري غصيلو ۽ ضد بنئيش(تيسو)وارو رويو ظاهر ڪندو آهي. (مثال ضد غصي ۾زمين تي ليٽي پوي يا شيون ٽوڙي) |
|  | گهڻو ڪري تنهائي پسند آهي، اڪيلو کيڏڻ پسند ڪندو آهي. |
|  | عام طور تي چيو مڃيندو آهي، جيڪو به وڏا ماڻهو چوندا اٿس ڪندو آهي. |
|  | گهڻيون پريشانيون اٿس، گهڻو ڪري پريشان رهي ٿو. |
|  | جيڪڏهن ڪنهن کي ڪا چوٽ لڳي، بيمار يا پريشان محسوس ڪري رهيو هجي ته ان جي مدد ڪندو آهي. |
|  | همشهه بي قرار رهي ٿو ۽ وٽ وڪڙ پيوکائيندو آهي.  (مثال ويٺي ويٺي به ٽنگيون ۽ ٻانهون پيو هلائيندو آهي) |
|  | هن جو گهٽ ۾ گهٽ هڪ سٺو دوست/سهيلي آهي.  (ڀيڻ ڀاءُ شامل نه ڪيو) |
|  | گهڻوڪري ٻين ٻارن سان وڙهي ٿو۽ڌمڪيون/ڌڙڪا ڏئي ٿو. |
|  | اڪثر ناخوش ۽ روئڻ هارڪورهي ٿو.(روئڻو آهي) |
|  | عام طور ٿي ٻيا ٻار هن کي پسند ڪن ٿا. |
|  | آساني سان توجهه هٽائي وڃي ٿو،مڪمل طور تي هٽائي ڇڏي ٿو۽گم(بيخيالو) ٿي وڃي ٿو. (مثال توهان ان کي ڪا ڳالهه چئو ۽ ان جوخيال ٻئي پاسي جلدي ٿي وڃي) |
|  | نئين ماحول۾گهبرائجي وڃي ٿو ۽ چنبڙي وڃي ٿو،اعتماد جلدي وڃائي ويهي ٿو. |
|  | ننڍن ٻارن سان رحمدل آهي. |
|  | گهڻو ڪري ڪوڙ ڳالهائي ٿويا دهوڪي بازي ڪري ٿو. |
|  | ٻيا ٻار هن کي تنگ ڪن ٿا يا ڌهمڪيون ڏين ٿا. |
|  | ٻين جي مدد جي لاء اڪثر پنهنجي اڳرائي پيش ڪري ٿو. (ماءُ، پيء، ڏاڏو ، ڏاڏي ، استاد، ٻيا گهر جا ڀاتي۽ ٻار). |
|  | عمل ڪرڻ سان پهريان غور ڪري ٿو.  (مثال مهمانن جي سامهون ڪابه حرڪت ڪرڻ کان پهريائين سوچي تي ڪرڻي آهي يا نه). |
|  | گهر، اسڪول يا ڪنهن ٻي جاء تان چوري ڪري ٿو. |
|  | ٻين ٻارن جي مقابلي ۾ وڏن ماڻهن سان بهتر دوستي ڪندو آهي . |
|  | گهڻو ڊڄڻو آهي ، آساني سان ڊڄي وڃي ٿو. |
|  | ڪم کي ختم ڪرڻ تائين نه ٿو ڇڏي ، توجه ڏيڻ جي مدت سٺي اٿس. (مثال دڪان تان ڪا شيء گهرائڻي ڏيوس ته ضرور وٺي اچي) |

Table C

*Correlations Between All Strengths and Difficulties Questionnaire Items*

| Item | 1 | 2 | 3 | 4 | 5 | 6 | 7 | 8 | 9 | 10 | 11 | 12 | 13 | 14 | 15 | 16 | 17 | 18 | 19 | 20 | 21 | 22 | 23 | 24 |
| --- | --- | --- | --- | --- | --- | --- | --- | --- | --- | --- | --- | --- | --- | --- | --- | --- | --- | --- | --- | --- | --- | --- | --- | --- |
| 1 | __ |  |  |  |  |  |  |  |  |  |  |  |  |  |  |  |  |  |  |  |  |  |  |  |
| 2 | -.04 | __ |  |  |  |  |  |  |  |  |  |  |  |  |  |  |  |  |  |  |  |  |  |  |
| 3 | -.01 | .02 | __ |  |  |  |  |  |  |  |  |  |  |  |  |  |  |  |  |  |  |  |  |  |
| 4 | .16 | -.12 | .09 | __ |  |  |  |  |  |  |  |  |  |  |  |  |  |  |  |  |  |  |  |  |
| 5 | -.11 | .29 | .11 | -.14 | __ |  |  |  |  |  |  |  |  |  |  |  |  |  |  |  |  |  |  |  |
| 6 | -.10 | -.04 | .05 | -.18 | .03 | __ |  |  |  |  |  |  |  |  |  |  |  |  |  |  |  |  |  |  |
| 7 | .17 | -.13 | .01 | .14 | -.14 | -.04 | __ |  |  |  |  |  |  |  |  |  |  |  |  |  |  |  |  |  |
| 8 | -.01 | .03 | .13 | -.04 | .07 | .06 | -.01 | __ |  |  |  |  |  |  |  |  |  |  |  |  |  |  |  |  |
| 9 | .28 | .01 | .04 | .14 | -.03 | -.03 | .15 | .04 | __ |  |  |  |  |  |  |  |  |  |  |  |  |  |  |  |
| 10 | -.03 | .39 | .02 | -.09 | .20 | .01 | -.08 | .10 | -.07 | __ |  |  |  |  |  |  |  |  |  |  |  |  |  |  |
| 11 | .20 | -.02 | .01 | .16 | -.04 | -.11 | .14 | .00 | .14 | -.04 | __ |  |  |  |  |  |  |  |  |  |  |  |  |  |
| 12 | -.12 | .29 | .06 | -.15 | .26 | .07 | -.18 | .07 | -.08 | .19 | -.06 | __ |  |  |  |  |  |  |  |  |  |  |  |  |
| 13 | -.03 | .13 | .14 | -.08 | .23 | .06 | -.13 | .21 | -.08 | .22 | -.05 | .18 | __ |  |  |  |  |  |  |  |  |  |  |  |
| 14 | .18 | -.03 | -.07 | .15 | -.11 | -.12 | .18 | -.07 | .19 | -.05 | .31 | -.14 | -.09 | __ |  |  |  |  |  |  |  |  |  |  |
| 15 | -.18 | .04 | .03 | -.02 | .06 | .04 | -.12 | .11 | -.15 | .13 | -.08 | .07 | .12 | -.18 | __ |  |  |  |  |  |  |  |  |  |
| 16 | -.06 | -.06 | .12 | -.05 | .08 | .07 | -.03 | .09 | -.07 | .04 | -.05 | .01 | .10 | -.06 | .13 | __ |  |  |  |  |  |  |  |  |
| 17 | .22 | -.07 | -.06 | .18 | -.10 | -.08 | .18 | -.04 | .19 | -.02 | .16 | -.19 | -.06 | .28 | -.06 | -.09 | __ |  |  |  |  |  |  |  |
| 18 | -.03 | .14 | .06 | -.05 | .14 | .05 | -.14 | .05 | -.07 | .13 | -.05 | .20 | .13 | -.05 | .09 | .00 | -.11 | __ |  |  |  |  |  |  |
| 19 | -.10 | .05 | .08 | -.04 | .08 | .04 | -.09 | .10 | -.11 | .12 | -.08 | .23 | .13 | -.11 | .14 | .11 | -.02 | .09 | __ |  |  |  |  |  |
| 20 | .13 | .09 | .03 | .06 | .06 | .01 | .11 | .03 | .19 | .05 | .15 | .05 | .02 | .14 | -.08 | -.03 | .14 | .02 | .07 | __ |  |  |  |  |
| 21 | -.15 | .17 | -.02 | -.12 | .11 | .01 | -.19 | -.03 | -.14 | .11 | -.08 | .18 | .10 | -.13 | .10 | -.02 | -.10 | .10 | .09 | -.02 | __ |  |  |  |
| 22 | -.16 | .06 | .10 | -.05 | .04 | .03 | -.05 | -.02 | -.06 | .00 | -.02 | .10 | -.01 | -.05 | .09 | .01 | -.09 | .23 | .11 | .03 | .09 | __ |  |  |
| 23 | .03 | .02 | .04 | .06 | .02 | .08 | .02 | .00 | .12 | -.02 | .04 | .01 | -.03 | .04 | -.01 | -.08 | .01 | .00 | -.05 | .10 | .01 | .00 | __ |  |
| 24 | -.03 | -.01 | .14 | -.01 | .05 | .06 | .01 | .16 | .02 | -.02 | -.03 | -.05 | .10 | -.02 | .05 | .26 | .01 | .02 | .16 | .04 | -.04 | .02 | .00 | __ |
| 25 | -.19 | .00 | .05 | -.04 | .06 | .02 | -.20 | -.01 | -.12 | .01 | -.16 | .05 | -.01 | -.23 | .16 | .08 | -.17 | .06 | .05 | -.15 | .12 | .08 | .00 | .05 |
