# Supplementary material for: Measuring and understanding social-emotional behaviors in preschoolers from rural Pakistan
Source: PLoS One. 2018 Nov 27;13(11):e0207807. doi: 10.1371/journal.pone.0207807 (PMC6258542; doi:10.1371/journal.pone.0207807)
Supplement: S3 Appendix — (DOCX) [file pone.0207807.s003.docx]

S3 Appendix

Final Items for Measures in English and Sindhi

Table A

*Final Items from the Self-Reported Questionnaire in Sindhi*

| **نمبر** | **سوال** |
| --- | --- |
| 01 | ڇا توهان کي اڪثر ڪري مٿي ۾ سور رهندو آهي؟ |
| 02 | ڇا توهان کي بک صحيح نه لڳي ٿي؟ |
| 03 | ڇا توهان صحيح نموني ننڊ نه ڪيو ٿا؟ |
| 04 | ڇا توهان آساني سان ڊڄي وڃو ٿا؟ |
| 05 | ڇا توهان جا هٿ ڏڪن ٿا؟ |
| 06 | ڇا توهان تمام گهڻي گهبراهٽ يا پريشاني محسوس ڪريو ٿا؟ |
| 07 | ڇا توهان جي هاضمي جو نظام ڪمزور / خراب آهي؟ |
| 08 | ڇا توهان کي صحيح سوچڻ ۾ مشڪل ٿئي ٿي؟ |
| 09 | ڇا توهان نا خوش محسوس ڪريو ٿا ؟ |
| 10 | ڇا توهان معمول کان وڌيڪ روئنديون آهيون؟ |
| 11 | ڇا توهان کي پنهنجي روز مره جي سرگرمين (ڪمن ) ۾ مزو وٺڻ ۾ مشڪل پيش اچي ٿي؟ |
| 12 | ڇا توهان کي فيصلا ڪرڻ ۾ مشڪل پيش اچي ٿي؟ |
| 13 | ڇا توهان جو روزانه جو ڪم متاثر ٿئي ٿو؟ |
| 14 | ڇا توهان زندگي ۾ بهتر انداز ۾ حصو وٺڻ جي قابل نه آهيو؟ |
| 15 | ڇا توهان جي شين ۾ دلچسپي ختم ٿي وئي آهي؟ |
| 16 | ڇا توهان محسوس ڪريو ٿا ته توهان ناڪارا ۽ بيڪار انسان آهيو؟ |
| 17 | ڇا توهان کي پنهنجي زندگي ختم ڪرڻ جو خيال ذهن ۾ رهندو آهي؟ |
| 18 | ڇا توهان سڄو ڏينهن ٿڪاوٽ محسوس ڪريو ٿا؟ |
| 19 | ڇا توهان کي پيٽ ۾ بي آرامده ڪيفيتون آهن؟ |
| 20 | ڇا توهان جلدي ٿڪجي پئو ٿا؟ |

Table B

*Final Items from the Home Observation of the Measurement Environment (48 months) in Sindhi*

| **I. LEARNING MATERIAL** |  |
| --- | --- |
| 1. ڇا ٻار وٽ ڪي اهڙا رانديڪا يا سکيا جو سامان آهي جيڪو توهان جي ٻارکي رنگ، شڪليون يا سائيز سيکارڻ ۾ استعمال ڪري سگهجي؟ ان ۾ گهريلو رانديڪا ياعام استعمال جا ٿانو به شامل آهن (چمچا ۽ ڪپ وغيره). **E** |  |
| 2. ڇا توهان ڪڏهن به ٻار لاءِ ڪو پزل (Puzzle) وارو رانديڪو ٺاهيو يا خريد ڪيو آهي؟ **E**  پزل يعني ڪا مجهاري واري يا گجهارت واري راند مثال ڪا تصوير ٽڪرن جي شڪل ۾ هجي جنهن کي جوڙي مڪمل ڪجي. |  |
| 3. ٻار وٽ ٽيپ، CD پليئر ۽ گهٽ ۾ گهٽ 5 ڪيسٽون يا سيڊيون آهن (موبائل فون تي ميوزڪ پڻ شامل آهي). **E** |  |
| 4. ٻار وٽ اهڙا رانديڪا ۽ گيم آهن جيڪي کليي اظهار جو موقعو ڏين (مثال مٽي جا رانديڪا پني جا رانديڪا ٻيڙي وغيره). **E** |  |
| 5. ٻار وٽ اهڙا رانديڪا آهن جنهن سان هو پيچيده چر پر ڪري سگهي. **E** |  |
| 6. ٻار وٽ اهڙا رانديڪا آهن جن سان انکي انگ سيکاري سگهجن (بلور ، ڪوڏيون، پٿريون ، موبائل فون گيم ،ڪيرم بورڊ يا عام استعمال جون شيون چمچا وغيرو). **E** |  |
| 7. ٻار کي گهٽ ۾ گهٽ 5 ڪتاب آهن ڀيڻ يا ڀائرن جا ڪتاب جيڪو ٻار کولي ڏسي ٿو قرآن مجيد کان علاوه. **E** |  |
| 8. گهٽ ۾ گهٽ 5 ڪتاب گهر ۾ نظر اچن ٿا ڀيڻ يا ڀائرن جا ڪتاب جيڪو ٻار کولي ڏسي ٿو قرآن مجيد کان علاوه. **E** |  |
| 9. ٻارگهر جي ڀاتين کي قرآن، مذهبي ڪتاب ۽ ٻيا ڪتاب ميگزين ۽ اخبارون وغيره پڙهندي روزانو ڏسي ٿو**؟ I** |  |
| 10. ٻارجي شڪليون سکڻ لاءِ حوصله افزائي ڪئي وڃي ٿي؟ **I** |  |
| **II. LANGUAGE STIMULATION** |  |
| 11 ٻار وٽ اهڙا رانديڪا موجود آهن جن جي ذريعي جانورن جا نالا سکي ٿو. سچ پچ جا جانور ڏسي سکي ته اهي به شامل ڪيو. **E** |  |
| 12. ٻار جي اکر سکڻ ۾ حوصله افزائي ڪئي وڃي ٿي.  (مثال: موبائل فون ، سليٽ ، پني يا زمين جي ذريعي ڀلي سنڌي ياانگريزي اکر هجن). **I** |  |
| 13 والدين ٻار کي سادا اخلاقي طريقا سيکارن ٿا. مثال مهرباني چوڻ، شڪريا، مونکي معاف ڪجو چوڻ، رشتن کي ادب سان پڪارڻ ۽ ڀيڻ، ڀاء کي ادي،ادو چوڻ وغيره. **I** |  |
| 14. ماء پئ ٻار کي ڳالهائڻ لاءِ همٿائن ٿا ۽ ٻار کي ٻڌڻ لاءِ وقت ڪڍن ٿا. **I** |  |
| 15. ٻار کي ناشتي يا مجهند جي ماني ۾ پنهجي پسند ٻڌائڻ جي اجازت آهي. **I** |  |
| 16. ماءُ پيء صحيح گرامر ۽ ضمير استعمال ڪن ٿا. **O** |  |
| 17. ماءُ پيء جو آواز ٻار لاءِ مثبت احساسات ٻڌائي ٿو. **O** |  |
| **III. PHYSICAL ENVIRONMENT** |  |
| 18 عمارت محفوظ ۽ خطرن کان خالي نظر اچي ٿي.. **O** |  |
| 19. ٻاهريون راند وارو ماحول محفوظ نظر اچي ٿو. **O** |  |
| 20. اندريون گهر اونداهون نه آهي. **O** |  |
| 21. آس پاس وارو ماحول خوبصورت ۽ خوشگوار آهي.  **O** |  |
| 22. گهر ۾ 100 چورس فوٽ في ماڻهو جڳهه آهي، اڱڻ به شامل ڪريو.  **O** |  |
| 23. ڪمرا سامان سان ڀريل نه آهن. **O** |  |
| 24. گهر صاف سٿرو آهي ۽ تڙيل پکڙيل نه آهي. **O** |  |
| **IV. RESPONSIVITY** |  |
| 25. ماءَ پئ ٻار کي 10 کان 15منٽ پنهنجي ويجهو جهلين ٿا. **I** |  |
| 26. وزٽ جي دوران ماءُ پئ گهٽ ۾ گهٽ ٻه دفعا ٻار سان ڳالهايو؟ **O** |  |
| 27. ماء پئ ٻار جي سوالن يا عرض جو زباني جواب ڏين ٿا. **O** |  |
| 28. ماء پئ عام طور تي ٻار جي گفتگو جو زباني جواب ڏين ٿا . **O** |  |
| **O**29. ماء پئ وزٽ جي دوران ٻه دفعا ٻار جي تعريف ڪئي. |  |
| 30. ماءِ پنهنجي ٻار کي پيار سان هٿ ڦيري ٿي ۽ان کي چميون ڏيئي ٿي يا ڀاڪر پائي ٿي. **O** |  |
| 31. وزٽ جي دروان ماءِ ٻار جي حوصله افزائي ڪري ٿي ته پنهنجون ڪجهه ڪاميابين جو مظارو ڪري ڏيکاري. **O** |  |
| **V. ACADEMIC STIMULATION** |  |
| 32. ٻار جي رنگ سکڻ ۾ حوصله افزائي ڪئي وڃي ٿي. **I** |  |
| 33. ٻار جي سُر واري ٻولي سکڻ ۾ حوصله افزائي ڪئي وڃي ٿي. (نظم ، بيت ، ڪلمو، دعا وغيره). **I** |  |
| 34. ٻارکي جاين سکڻ ۾حوصله افزائي ڪئي وڃي ٿي(هيٺان،مٿان، اندر،ٻاهر وغيره). **I** |  |
| 35. ٻارجي انگ سکڻ ۾ حوصله افزائي ڪئي وڃي ٿي. **I** |  |
| 36. ٻار جي ڪجهه لفظ سکڻ ۽ پڙهڻ ۾ حوصله افزائي ڪئي وڃي ٿي. **I** |  |
| **VI. MODELING** |  |
| 37. ٻار کاڌي ۾ ڪجهه دير برداشت ڪري ٿو. **I** |  |
| 38. ٻار کي ٽي وي قانون سان ڏيکاري وڃي ٿي. **I** |  |
| 39. ٻار خراب ردعمل کانسواءِ پنهنجي منفي جذبات جواظهار ڪري سگهي ٿو . **I** |  |
| 40.ٻارخراب ردعمل کانسواءِ ماءِ پيءِکي ڌڪ هڻي سگهي ٿو. **I** |  |
| 41. ماءِ پيء وزيٽر سان ٻار جو تعارف ڪرائن ٿا. **O** |  |
| **VII. VARIETY** |  |
| 42. ٻاروٽ سچو يا رانديڪي وارو موسيقي وارواوزار آهي. **E** |  |
| 43. ٻارکي گهٽ ۾گهٽ ٻن هفتن۾ ٻاهر گهمائڻ لاءِوٺي ويندا آهيو. **I** |  |
| 44. ٻار کي گذريل سال تقريبا هڪ دفعو 80 kmپري گهمائڻ لاءِ وٺي ويا هئا. **I** |  |
| 45. ٻار کي گذريل سال ۾ عجائب گهر وٺي ويا آهيو. **I** |  |
| 46. ماء پي ٻار کي پنهنجا رانديڪا بغير ڪنهنجي مدد جي کڻي رکڻ لاءِ حوصله افزائي ڪن ٿا. **I** |  |
| 47. ٻار گهٽ ۾ گهٽ هڪ ڀيرو يا اڪثر ڪري ماء يا پئ سان گڏ کاڌو کائي ٿو. **I** |  |
| 48. ماءِ پئ ٻارکي دڪان تي ڪي خاص کاڌا چونڊڻ جي اجازت ڏين ٿا. **I** |  |
| 49. ماءُ پي ڏکيا جملاءِ ،صحيح لغت استعمال ڪن ٿا. **O** |  |
| 50. گهر ۾ ڪنهن جاءِ تي ٻار جو فن وارو ڪم ظاهر ٿيل آهي**O**. |  |
| **VIII. ACCEPTANCE** |  |
| 51. گذريل هفتي ۾ هڪ کان وڏيڪ دفعا جسماني سزا جو واقعو نه ٿيو آهي**I** . |  |
| 52. وزٽ جي دوران ماء پئ ٻار تي هڪ کان وڌيڪ ڀيرا ڪاوڙ ، ڦٽڪار نه ڪئي. **O** |  |
| 53. وزٽ جي دوران ماء پئ جسماني ڌتڪار نه ڪئي**O**. |  |
| 54. وزٽ جي دوران ماءُ پئ ٻار کي چماٽ يا ٿڦڙ نه لڳايو. **O** |  |
